# Investigation of Fibrillar Aggregates Formed by Pathogenic Pre-pro-vasopressin Mutants that Cause ADNDI

**DOI:** 10.1210/clinem/dgae749

**Published:** 2024-10-25

**Authors:** Refika Dilara Vaizoglu, Beril Erdem, Mehmet Gul, Ceren Acar, Huseyin Ozgur Ozdemirel, Emel Saglar Ozer, Hatice Mergen

**Affiliations:** Department of Biology, Universities District, Hacettepe University, Faculty of Science, Molecular Biology Section, Beytepe Campus, Cankaya/Ankara 06800, Turkey; Department of Biology, Universities District, Hacettepe University, Faculty of Science, Molecular Biology Section, Beytepe Campus, Cankaya/Ankara 06800, Turkey; Inonu University, Faculty of Medicine, Basic Medical Sciences, Histology and Embryology Section, Malatya 44280, Turkey; Department of Molecular Biology and Genetics, Inonu University, Faculty of Arts and Science, Malatya 44280, Turkey; Department of Biology, Universities District, Hacettepe University, Faculty of Science, Molecular Biology Section, Beytepe Campus, Cankaya/Ankara 06800, Turkey; Department of Biology, Universities District, Hacettepe University, Faculty of Science, Molecular Biology Section, Beytepe Campus, Cankaya/Ankara 06800, Turkey; Department of Biology, Universities District, Hacettepe University, Faculty of Science, Molecular Biology Section, Beytepe Campus, Cankaya/Ankara 06800, Turkey

**Keywords:** aggregates, ADNDI, *AVP-NPII*, amyloid, neurodegenerative diseases

## Abstract

**Context:**

Aggregations of unfolded or misfolded proteins, both inside and outside cells, are implicated in numerous diseases, collectively known as amyloidosis. Particularly, autosomal dominant neurohypophyseal diabetes insipidus (ADNDI) is a rare disease caused by mutations in the *AVP-NPII* gene, leading to the inability to secrete arginine vasopressin. These misfolded proteins accumulate within the endoplasmic reticulum (ER), causing cellular dysfunction.

**Objective:**

This study aimed to investigate the formation of amyloid-like aggregates within the cell resulting from misfolded mutant precursor proteins, which induce disulfide-linked oligomers due to the G45C, 207_209delGGC, G88V, C98X, C104F, E108D-1, E108D-2 and R122H mutations identified by our group in the *AVP-NPII* gene of ADNDI patients.

**Methods:**

Deglycosylation studies were performed to analyze the glycosylation patterns of mutant protein precursors. The involvement of these precursors in the ER-related degradation pathway was studied by conducting protease inhibition experiments. Disulfide-linked oligomer analysis determined the oligomerization status of the mutant precursors. Immunofluorescence and electron microscopy studies provided evidence of aggregate structures in the ER lumen. In vitro studies involved bacterial expression and fibril formation in *Escherichia coli* (*E. coli*).

**Results:**

Our findings demonstrated that the N-glycan structure of mutant precursors remains intact within the ER. Protease inhibition experiments indicated the involvement of these precursors in the ER-related degradation pathway. Disulfide-linked oligomer analysis revealed homo-oligomer structures in mutations. Immunofluorescence and electron microscopy studies confirmed the presence of aggregate structures in the ER lumen. In vitro studies showed that mutant precursors could form fibril structures in *E. coli*.

**Conclusion:**

Our study may support the idea that ADNDI belongs to the group of neurodegenerative diseases due to the formation of fibrillar amyloid aggregates in the cell.

Autosomal dominant neurohypophyseal diabetes insipidus (ADNDI) is a disease caused by the lack of the secretion of arginine vasopressin (AVP) which has an important role in regulating water balance of the human body. The disease is characterized by polydipsia and polyuria, which can be seen a few months after birth (1, 2). The antidiuretic hormone AVP is synthesized in magnocellular neurons of the supraoptic and paraventricular nuclei of the hypothalamus, and mutations in the AVP gene are responsible for ADNDI. AVP locates on chromosome 20, which encodes the human precursor hormone pre-pro-AVP. Precursor protein is 164 amino acid-length and consists of N-terminal signal peptide, AVP, neurophysin II (NPII), and C-terminal copeptide structure. After the synthesis of pre-pro-AVP precursor protein in the nucleus of neuronal cells, it is translocated to the ER lumen by the N-terminal signal peptide. When the precursor protein enters the ER translocon, the signal peptide sequence is removed by signal peptidases. Precursor protein folds with molecular chaperones, and pro-AVP is formed by attaching the N-glycan structure to the C-terminal copeptin. This conformation is stabilized with 8 disulfide bridges between the cysteine residues. The prohormone is transmitted along the axon in vesicles and stored in the posterior pituitary until it is secreted by potential exchange within the nerve terminals (3, 4).

More than 83 mutations in the *AVP* gene are located in signal peptide; AVP and NPII have been reported as linked with ADNDI (4). Until now, no mutation has been found in the copeptid sequence. Studies with mutant AVP precursors have shown that the misfolded mutant prohormone causes defects in the intracellular transportation of protein (2). Studies have shown that dominant mutations in the *AVP* gene cause mutant precursors that form disulfide-linked homo-oligomer structures, and consequently aggregation occurs in the ER lumen. These results support the assignment of ADNDI disease to the group of neurodegenerative diseases associated with the formation of fibrillary amyloid aggregates (5-8).

In this work, we performed intracellular traffic analysis and localization of aggregate structures of mutant precursors caused by G45C, 207_209delGGC, G88V, C98X, C104F, E108D-1, E108D-2, and R122H mutations in the *AVP* gene, which were also previously identified in Turkish patients by our studies (9-14). Here we showed that misfolded mutant precursor proteins formed amyloid-like aggregates in the lumen of ER. In addition, it was found that all mutant precursors formed disulfide-linked oligomers; they were retranslocated by the ER-related degradation pathway and formed aggregate structures, which were shown within the ER lumen by immunofluorescence and electron microscopy. Also, the in vitro fibril formation abilities of mutant precursor proteins were determined by using *Escherichia coli*.

## Materials and Methods

### Generation of Mutant AVP Constructs

G45C, 207_209delGGC, G88V, C98X, C104F, E108D-1, E108D-2, and R122H mutations were introduced into the pLAVP construct, which contains the coding sequence for the human AVP with an N-terminal HA-tag and C-terminal FLAG-tag. pLAVP construct had previously been employed by our group for the functional analysis of mutant AVPs (10). To generate these mutations, PCR-based site-directed mutagenesis with restriction fragment exchange strategy, along with the In-Fusion Snap Assembly Cloning Kit (TaKaRa, Japan), were utilized.

For the expression of mutant AVPs in bacteria, C-terminal 6-His-tagged pET21b expression vector was used, and the same mutations were generated using PCR-based site-directed mutagenesis and restriction fragment exchange strategy. All mutant constructs were confirmed by DNA sequencing.

### Cell Culture and Transfection Studies

COS-1 (ATCC, Cat# CRL-1650, RRID:CVCL_0223) cells were cultured in DMEM (Sigma-Aldrich, USA) supplemented with 10% fetal bovine serum (FBS), 100 U/mL penicillin, 100 µg/mL streptomycin, and 2 mM L-glutamine at 37 °C in a 5% CO_2_ incubator. For deglycosylation, protease inhibition, electron microscopy studies, and analysis of disulfide-linked oligomerization, cells were cultured in 6-well plates (400 000 cells/well, 2 mL). Immunofluorescence studies were conducted in 24-well plates (90 000 cells/well, 100 µL). To enhance production of recombinant proteins, cells were incubated for 24 hours before the transient transfection with 1 mM valproic acid (SERVA Electrophoresis GmbH, USA) in DMEM supplemented with 3% FBS, 100 units/mL penicillin, 100 µg/mL streptomycin, and 2 mM L-glutamine. Transient transfection was performed using TurboFect™ Transfection Reagent (Thermo Fisher Scientific). After 48 hours of incubation, plasmid coding enhanced green fluorescence protein was used to assess the transfection efficiency.

### Immunoblotting Studies

After the transfection, cells were washed with cold PBS, then lysed and scrapped with 500 µL lysis buffer [PBS, 1% Triton X-100, 0.5% Na-deoxycholate, 2 mM phenylmethylsulfonyl fluoride (PMSF), 250 U/mL benzonase]. Cell lysates were incubated on ice for 30 minutes and then centrifuged at 13 000 rpm for 30 minutes at 4 °C. Total protein concentrations were measured using the Bradford protein assay. The total protein samples were mixed with loading dye containing β-mercaptoethanol (BioRad, USA) and denatured at 65 °C for 10 minutes. Approximately 30 μg total protein was loaded onto a 12.5% SDS-PAGE. Protein samples were transferred to a polyvinylidene difluoride mini membrane (Amersham™, USA) using the Trans-Blot Turbo Transfer system (BioRad). The membrane was immunolabeled using horseradish peroxidase conjugated anti-HA-Tag (6E2) mouse mAB (Cell Signaling Technology, Cat# 2999, RRID:AB_1264166) antibody at a dilution of 1:1000. Then, the immunolabel membrane was covered with Clarity Max™ Western ECL Substrate (BioRad) and visualization was performed using the ChemiDoc™ imaging system (BioRad).

### De-glycosylation Studies

After isolating the total protein, for endoglycosidase H (Endo H; New England BioLabs, UK) digestion, all samples were boiled for 10 minutes with 10X glycoprotein denaturation buffer (New England BioLabs) according to the manufacturer's protocol. Then they were incubated with 10X Glikobuffer 3 and 500-unit Endo H for 2 hours at 37 °C. For peptide N glycosidase F (PNGase F; New England BioLabs) digestion, protein samples were incubated with 10X Glikobuffer 2 containing 10% NP-40 and 500-unit PNGase F (New England BioLabs) for 2 hours at 37 °C. After enzyme digestions, the samples were immunoblotted as described previously.

### Analysis of Disulfide-linked Oligomers

Cells were incubated with iodoacetamide (SERVA Electrophoresis GmbH) to alkylate the free sulfhydryl groups of the proteins and prevent oxidation after lysis. After 48 hours of transfection, cells were washed with cold PBS containing 100 mM iodoacetamide and incubated with 200 mM Tris (pH 8.0, contains 100 mM iodoacetamide, 2 mM PMSF) at 4 °C for 1 hour in the dark. After incubation, cells were scrapped and lysed in lysis buffer (PBS, 1% Triton X-100, 0.5% Na-deoxycholate, 2 mM PMSF 250 U/mL benzonase with 100 mM iodoacetamide). For immunoblotting experiments, one-half of each total protein sample was reduced with β-mercaptoethanol in loading dye, and the other half was left unreduced. Reduced and unreduced protein samples were run on sodium dodecyl sulfate (SDS) gel, as described in the immunoblotting section. For the analysis of the oligomerization type of mutants, the lane containing an unreduced sample on the SDS gel was cut out and reduced with 200 mM β-mercaptoethanol in loading dye. The gel strip was then applied horizontally on a second SDS gel and analyzed by immunoblotting.

### Protease Inhibition Studies

After the transfection, cells were incubated with proteasomal inhibitor E-64 (Cayman Chemical Company, USA) at a concentration of 250 μM in DMEM (supplemented with 10% FBS, 100 U/mL penicillin, 100 µg/mL streptomycin, and 2 mM L-glutamine) for 90 minutes prior to the experiment. After the incubation period, cells were lysed and scrapped with lysis buffer (PBS, 1% Triton X-100, 0.5% Na-deoxycholate, 2 mM PMSF, 250 U/mL benzonase). Total protein concentrations were measured using the Bradford protein assay. Samples were then analyzed by immunoblotting, following the procedure described previously.

### Immunofluorescence Microscopy Studies

COS-1 cells were cultured on poly-D-lysine (Santa Cruz Biotechnology, USA)-coated glass coverslips in 24-well plates, as described earlier. To visualize the type of aggregate structure, cells were transiently cotransfected with both mutant AVPs and segrotagranin II MYC. After 48 hours transfection, cells were fixed with 3% paraformaldehyde (SERVA Electrophoresis GmbH), permeabilized with 0.1% Tween-20 (SERVA Electrophoresis GmbH) in PBS for 30 minutes at 37 °C and blocked with 0.1% Tween-20, 20 mM Glisin (SERVA Electrophoresis GmbH) and 1% BSA (SERVA Electrophoresis GmbH) in PBS for 20 minutes at 37 °C. Rabbit anti-human AVP polyclonal antibody (MyBioSource, Cat# MBS9205129, RRID:AB_3572259, 1:50) primer antibody and Goat Anti-Rabbit IgG H&L Alexa Fluor®488 (Abcam Cat# ab150077, RRID:AB_2630356 1:1000) secondary antibody were used for wild-type and mutant AVPs. Myc-Tag (E7F98) Mouse mAb (Cell Signaling Technology, Cat# 92013, RRID:AB_2800176 1:200) primer antibody and Goat Anti-Rabbit IgG H&L Alexa Fluor®555 (Abcam, Cat# ab150114, RRID:AB_2687594 1:1000) secondary antibody were used for segrotagranin protein. Additionally, to visualize whether the aggregates accumulated in ER or not, cells were transiently transfected with mutant AVPs and anti-AVP and Myc-Tag (E7F98) Mouse mAb (Cell Signaling Technology Cat# 92013, RRID:AB_2800176 1:200) antibodies were used as described previously. For the detection of ER, anti-BAP31 antibody (7A3BB6) (Abcam, Cat# ab112993, RRID:AB_10864916 5 µg/mL) primary antibody, which showed specific reactivity to the BAP31 protein in the ER lumen Goat Anti-Rabbit IgG H&L Alexa Fluor®555 (Abcam Cat# ab150114, RRID:AB_2687594 1:1000) secondary antibody, were used. After incubation with secondary antibodies, cells were washed with 0.1% Tween-20 in PBS and coverslips were mounted on glass slides using Mowiol®4-88 (Sigma-Aldrich). Cell images were captured using a confocal laser scanning microscope at the Microscopy Laboratory, Central Laboratory, Molecular Biology and Biotechnology R&D Center of METU (Zeiss LSM 510).

### Electron Microscopy Studies

After transient transfection of cells with mutant AVPs, they were harvested and fixed with a solution containing 3% paraformaldehyde and 2.5% glutaraldehyde (SERVA Electrophoresis GmbH) in PBS. Cells were then permeabilized with 0.1% Tween-20 in PBS for 30 minutes and blocking was performed with 3% BSA in PBS. Subsequently, cells were incubated with rabbit anti-human AVP polyclonal antibody (MyBioSource Cat# MBS9205129, RRID:AB_3572259, 1:50) as a primary antibody for 3 hours at room temperature with gentle rotation at 20 rpm. As a secondary antibody, Goat pAb to Rb IgG Gold 15 nm (Abcam Cat# ab27236, RRID:AB_954457 1:1000) was applied overnight at 4 °C with gentle rotation at 20 rpm. Afterward, cells were incubated with a 1% osmium tetroxide (SERVA Electrophoresis GmbH) solution at 4 °C for 1 hour and washed with PBS. The cells were dehydrated and embedded in epoxy araldite (Agar Scientific, UK) according to the manufacturer's instructions and polymerized at 60 °C for 3 days. The polymerized araldite cell blocks were trimmed using the trimer instrument (Leica EM TRIM). Semi-thin sections were obtained from the trimmed araldite cell blocks using with an ultramicrotome (Leica Ultracut R microtome) to prepare ultra-thin sections. Ultra-thin sections were mounted on copper-coated formvar (Electron Microscopy Sciences, USA) and visualized using a transmissive electron microscope (TEM; Zeiss Libra 120).

### Bacterial Expression and In Vitro Fibril Formation Studies

Bacterial transformation experiments were performed to express mutant AVPs in *Escherichia coli* BL21(DE3). Bacterial cells were incubated with 1 mM isopropyl-D-thiogalactoside (SERVA Electrophoresis GmbH) for 2 to 3 hours 37 °C with shaking at 180 rpm. The Ni-NTA chromatography kit (Qiagen, Germany) was utilized to isolate the proteins from bacteria following the manufacturer's protocol. Subsequently, the proteins were dialyzed using a Slide-A-Lyzer™ G2 dialysis cassette (Thermo Fisher Scientific). After dialysis samples were applied onto a copper-coated Formvar grid (Electron Microscopy Sciences) and allowed to dry. The dried samples were then stained with 1% uranyl acetate (Agar Scientific) and images were captured with a TEM (Zeiss Libra 120).

## Results

### Analysis of Glycosylation Patterns in Mutant Protein Precursors

**Figure 1.**
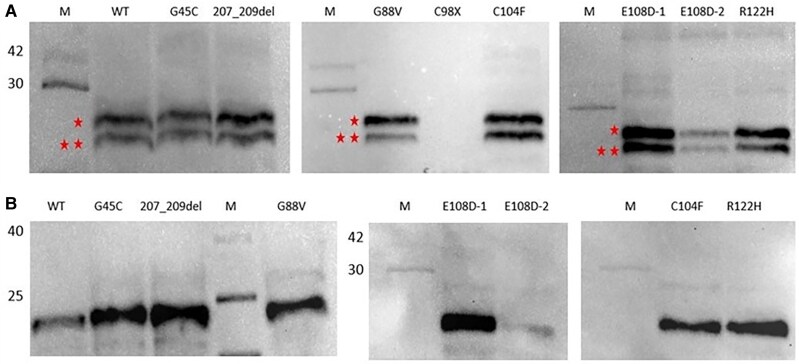
Total protein and deglycosylation studies. (A) The SDS-PAGE image of wild-type and mutant AVP precursors isolated from COS-1 cells. The band indicated by a single asterisk represents glycosylated pre-pro-AVP with an approximate molecular weight of 27 kDa, while the band indicated by a double asterisk represent nonglycosylated pre-pro-AVP with an approximate molecular weight of 21 kDa. (B) The SDS-PAGE image of wild-type and mutant AVP precursors from transient transfected COS-1 cells after endoglycosidase H digestion. The bands are approximately 27 kDa in size. Abbreviations: AVP, arginine vasopressin.

The protein analysis results revealed the presence of a glycosylated pre-pro-AVP of 27 kDa and a nonglycosylated pre-pro-AVP of 21 kDa, as shown in Fig. 1A. The glycosylation status of the mutant pre-pro-AVPs was examined by treating them with Endo H and PNGase F enzymes. Upon enzymatic treatment, the high mannose structure was removed from the glycosylated protein of approximately 27 kDa in both the wild-type and mutant proteins. Additionally, the mutant proteins lacked the high mannose structure and showed no changes upon treatment with the PNGase F enzyme. The immunoblot analysis did not detect the C98X (stop codon) mutant protein (Fig. 1B).

### Protease Inhibition Results

**Figure 2.**
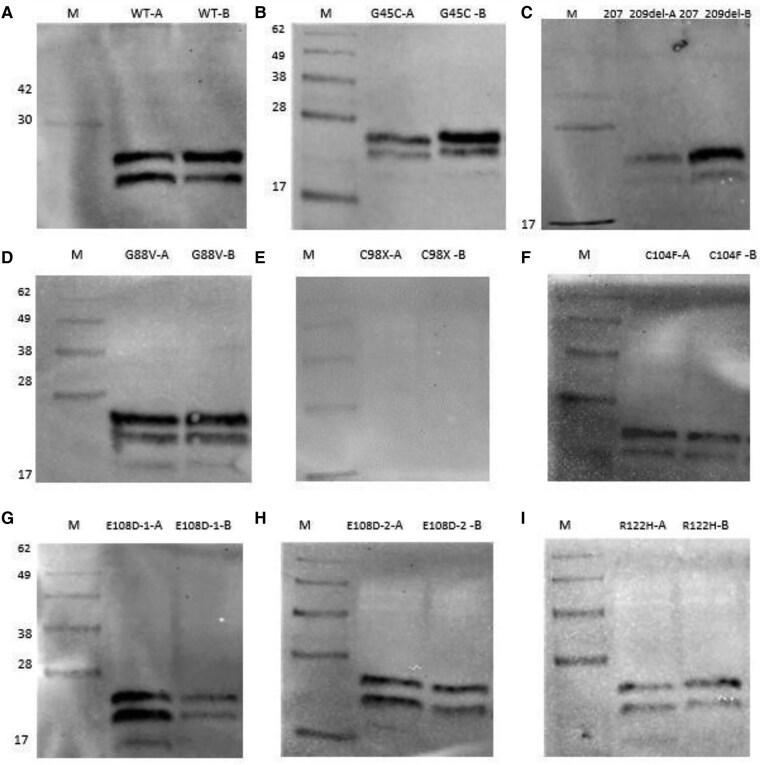
Protease inhibition images. The SDS-PAGE image of pre-pro-AVP. The bands indicate glycosylated pre-pro-AVP at approximately 27 kDa and nonglycosylated pre-pro-AVP at 21 kDa. No degradation products were observed. (A) Control group without protease inhibitors; (B) E-64 protease inhibitor applied. Abbreviations: AVP, arginine vasopressin.

The first group of cells designated as the control group were not treated with a protease inhibitor, and as shown in the figure, both glycosylated and nonglycosylated mutant proteins were observed. In the second group of cells, the proteasomal inhibitor E-64 was added to investigate the ER-associated degradation pathway. Immunoblot analysis revealed that AVP precursor proteins preferred the ER-associated degradation pathway (Fig. 2).

### Analysis of Disulfide-linked Oligomers

**Figure 3.**
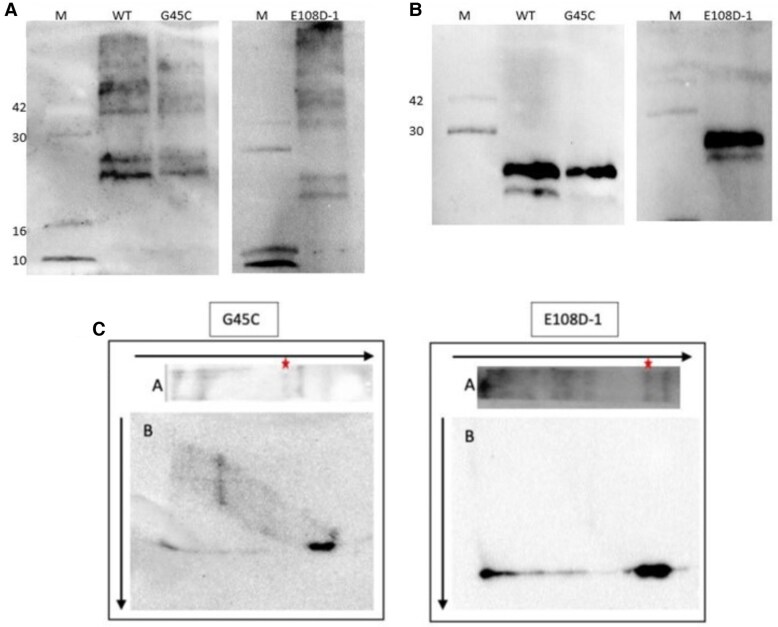
Analysis of disulfide-linked oligomers. (A) SDS-PAGE image of proteins treated with iodoacetamide under nonreducing conditions. Bands at approximately 27 kDa indicate glycosylated pre-pro-AVP, and bands at 21 kDa represent nonglycosylated pre-pro-AVP. (B) SDS-PAGE image of proteins treated with iodoacetamide under reducing conditions (SDS loading dye containing beta-mercaptoethanol). Bands at approximately 27 kDa indicate glycosylated pre-pro-AVP, and bands at 21 kDa represent nonglycosylated pre-pro-AVP. (C) Mutant AVP samples run in nonreducing SDS gel (A) were excised from the gel and subjected to reduction with β-mercaptoethanol and then placed horizontally on a second SDS gel (B). Two mutant AVP samples (G45C and E108D-1) that provided the best images on the nonreducing SDS gel were selected for the second separation. Arrows indicate the direction of gel migration. The star indicates glycosylated pre-pro-AVP at approximately 27 kDa. In the first gel (A), larger disulfide-bound oligomers above the 27 kDa band confirm the formation of homo-oligomers. On the second gel (B), some oligomer bands are closer to each other, resulting in a more circular band shape, and the band boundaries are not distinct due to the proximity of the bands on the first gel (A). Abbreviations: AVP, arginine vasopressin; SDS, sodium dodecyl sulfate.

The disulfide-linked oligomer formation of wild-type and mutant AVP precursors was examined. Under reducing conditions, both wild-type and mutant AVP precursors were detected as 27 kDa glycosylated and 21 kDa non-glycosylated pre-pro AVP, as shown in Fig. 3A. In the second step, the iodoacetamide-treated proteins were mixed with loading dye without 2-mercaptoethanol under nonreducing conditions, resulting in low-size protein bands (Fig. 3B). In the third step, a second dimensional analysis was performed to determine the homo- or hetero-oligomer status of the disulfide-linked oligomers obtained under nonreducing conditions. The results revealed that the G45C and E108D-1 mutations formed homo-oligomers in the form of a single band (Fig. 3C).

### Immunofluorescence Microscopy Results

**Figure 4.**
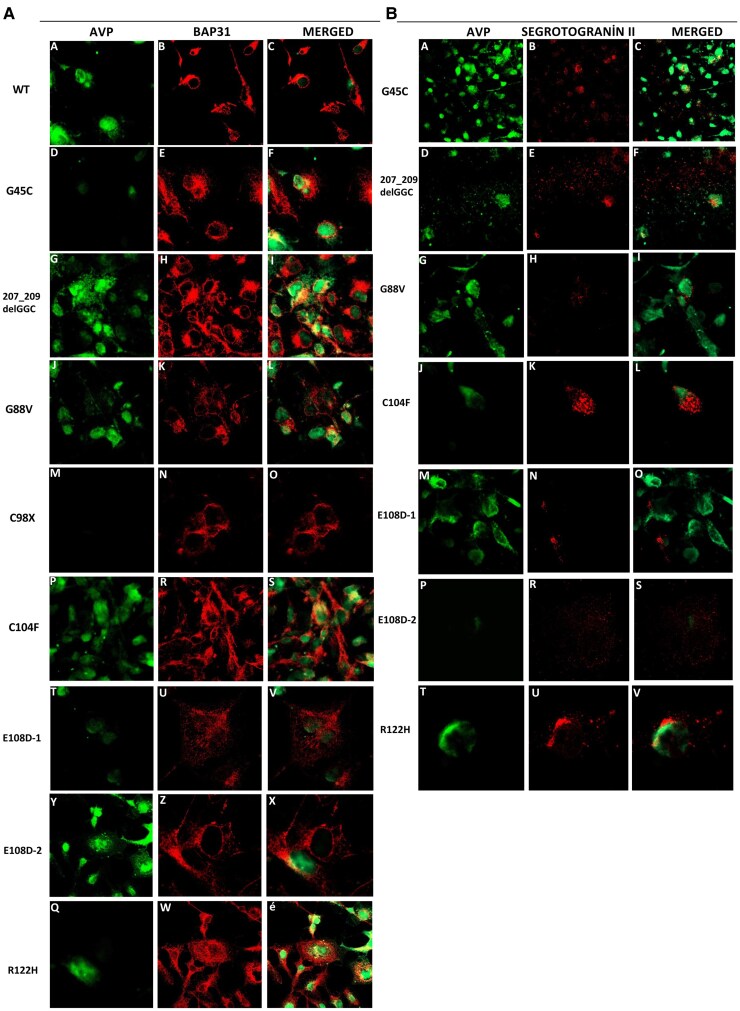
Immunofluorescence images. (A) Immunofluorescence images of wild-type and mutant protein precursors. Mutant proteins were captured using primary antibodies: anti-AVP antibody rabbit anti-human AVP polyclonal antibody (1:50) from Mybiosource, and the ER luminal protein BAP31 was captured using anti-BAP31 antibody [7A3BB6] (5 ug/mL) from Abcam. Secondary antibodies used were Goat Anti-Mouse IgG H&L Alexa Fluor 555 (red) from Abcam and Goat Anti-Rabbit IgG H&L Alexa Fluor 488 (green) from Abcam. (B) Immunofluorescence images of mutant protein precursors and granule-like structures. Mutant proteins were captured using primary antibodies (Anti-AVP antibody Rabbit anti-Human AVP polyclonal Antibody (1:50) from Mybiosource), and segrotogranin II-MYC was captured using Myc-Taq Mouse mAb (9B11) (1:200) from CST, labeled with Alexa Fluor 555. Secondary antibodies used were Goat Anti-Mouse IgG H&L Alexa Fluor 555 from Abcam and Goat Anti-Rabbit IgG H&L Alexa Fluor 488 from Abcam. Abbreviations: AVP, arginine vasopressin; ER, endoplasmic reticulum.

Wild-type and mutant AVPs' intracellular localization was determined by staining with fluorescent-labeled antibodies (Fig. 4A). Cells were cotransfected with chromogranin II, a protein known for forming granule structures within the cell, to enable compare of aggregate structures (Fig. 4B).

### Electron Microscopy Results

**Figure 5.**
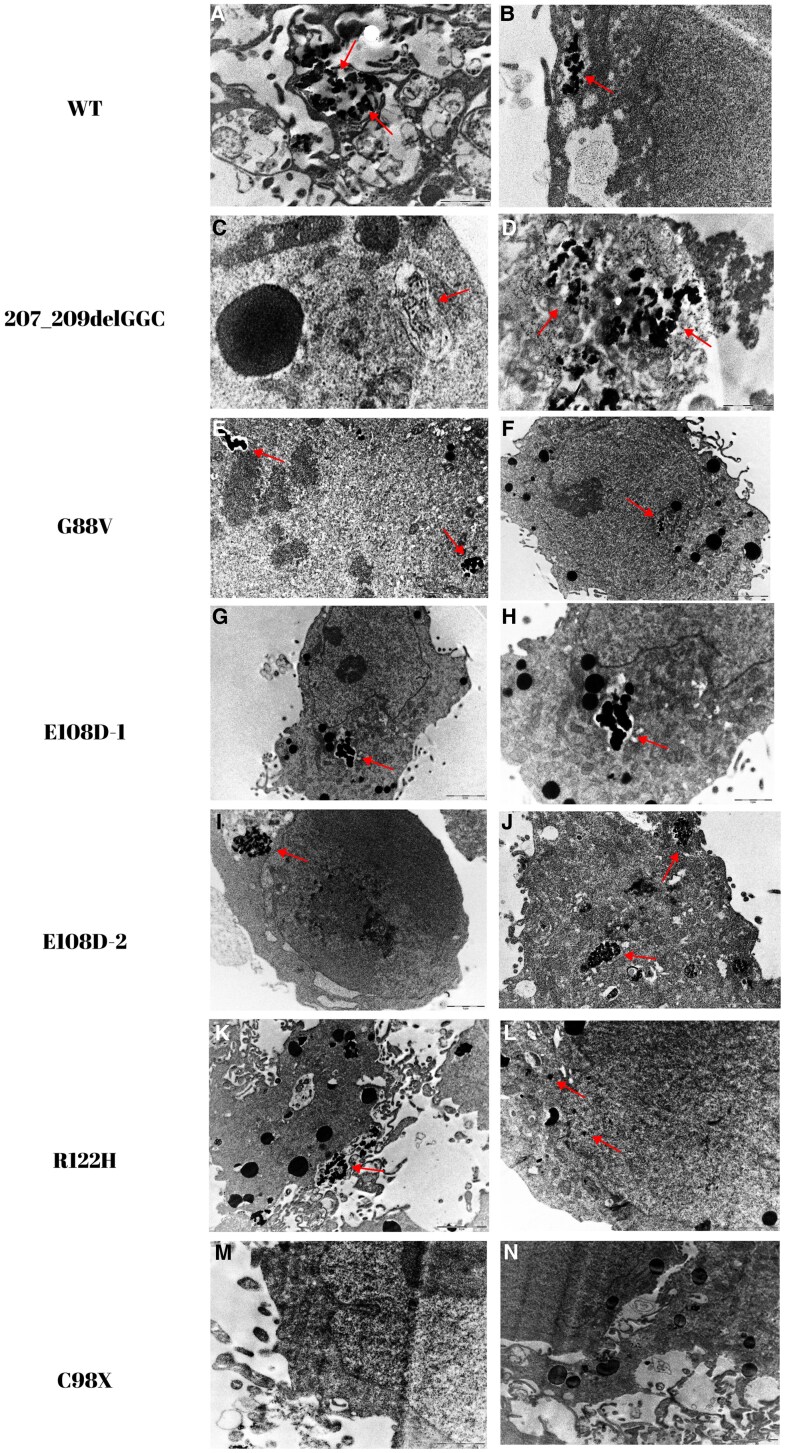
Electron microscopy images. Images showing electron-dense aggregate structures within intracytoplasmic and exocytic vesicles. For the wild-type sample, the red arrow indicates the secreted pro-AVP. For the 207_209delGGC, G88V, E108D-1, E108D-2, and R122H mutations, the red arrow indicates electron-dense protein aggregate structures. The image on the right side of the E108D-1 mutation is a zoomed-in version of the image on the left side. However, in the case of the C98X mutation, no protein expression was observed due to the stop codon. The images were obtained at scales of 1 µm, 2 µm, and 200 nm.

The intracellular localization and aggregate structures of mutant precursors, labeled for identification, were investigated using a TEM (Zeiss Libra 120). The aggregate structures formed are shown in the ER lumen (except for C98X stop codon mutant) (Fig. 5).

### In Vitro Fibril Formation Studies Results

**Figure 6.**
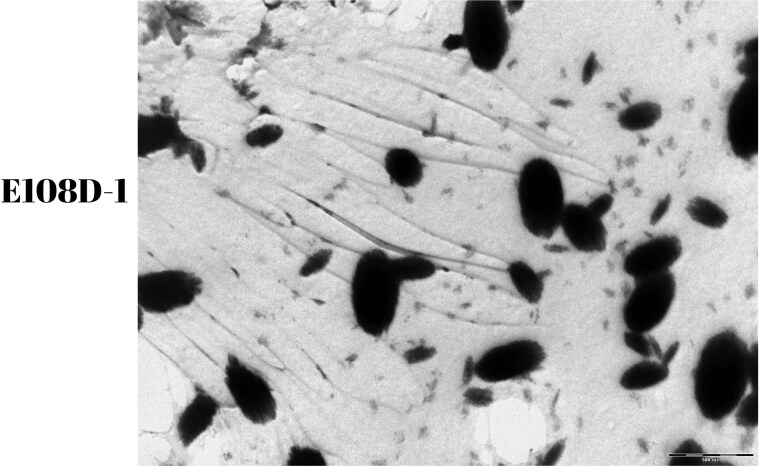
Bacterial fibril images. The electron microscopy images of fibril structures formed because of bacterial expression of the E108D-1 mutant protein precursor. The images display the characteristic fibril formation of the mutant protein in the bacterial system.

The collected samples were visualized using a TEM (Zeiss Libra 120). Mutant AVP precursors were observed to spontaneously formed fibrils under oxidizing conditions (Fig. 6).

## Discussion

The AVP hormone, also known as arginine vasopressin, plays a crucial role in regulating water balance in the body. Mutations in the gene responsible for encoding the AVP protein can lead to reduced or complete absence of hormone secretion. This condition is associated with impaired urine concentration in the kidneys and is characterized by excessive thirst and production of dilute urine, known as ADNDI. Mutations such as point mutations, deletions, or those introducing premature stop codons in the coding gene can result in the misfolding of AVP proteins and progressive degeneration of vasopressinergic neurons in the ER. Previous studies have identified that most of these mutations are located in the NPII region of the *AVP* gene. This region plays a critical role in the synthesis, proper folding, and transportation of AVP within the body (3, 15).

Upon entering the ER lumen, most proteins undergo posttranslational modifications, including glycosylation, as they are targeted to various cellular organelles, the plasma membrane, or the extracellular space. Once inside the ER, these proteins pass through ER-associated degradation (ERAD) checkpoints, where N-glycan structures are attached. Subsequently, they are transferred to the Golgi apparatus for further processing. Within the Golgi apparatus, protein maturation continues with the addition of high-mannose structures. The progression of this intracellular maturation traffic can be assessed through the activities of specific enzymes, such as Endo H, which removes N-glycan structures, and PNAGase-F, which eliminates high-mannose structures. The specialized enzyme involved in N-glycan removal alters the susceptibility of the enzymes responsible for cleaving either the entire sugar chain or individual monosaccharides of the N-glycan. As a result, protein bands of varying sizes can be observed by SDS-PAGE analysis (16).

In this study, deglycosylation analyses were conducted to investigate the intracellular trafficking of mutant precursors. It was observed that both the wild-type and all mutant precursors showed sensitivity to the Endo H enzyme, resulting in the removal of the glycan structure when treated with this enzyme. Based on this, we hypothesized that mutant pre-pro-AVP precursors enter the ER and undergo N-glycan attachment (Fig. 1B). However, when treated with the PNGase F enzyme, no noticeable changes or SDS-PAGE results were obtained for the mutant pre-pro-AVP precursors. This outcome was due to the inability of the mutant precursors to possess high-mannose structures, indicating that the protein cannot pass through the ER to the Golgi apparatus. Christensen et al demonstrated similar findings in which they investigated the glycosylation status of prohormones in both cell lysates and cell culture media by performing immunoprecipitation of cells. The results revealed that the cell lysates obtained after immunoprecipitation were sensitive to Endo H enzyme, indicating the presence of glycosylated prohormones in the cell lysates. On the other hand, the results obtained from immunoprecipitation of the cell culture media showed resistance to the enzyme, suggesting that glycosylated prohormones undergo modification upon reaching the Golgi complex (17).

Mutant precursors that accumulate within the ER lumen are degraded by proteasomes via ER-associated degradation and subsequently retranslocated into the cytosol. Protease inhibition studies have shown that these mutant precursors, when accumulated within the ER lumen, remain stable in the presence of proteasomal inhibitors. In studies focused on mutant precursors, excluding those with the C98X mutation (stop codon), a cysteine protease inhibitor (E-64) was used to observe proteosomal degradation. This inhibitor irreversibly binds to the active sites of cysteine proteases, thereby inhibiting them, and is commonly used for monitoring cellular events such as protein degradation and apoptosis. In our study, SDS-PAGE analysis demonstrated that in the presence of inhibitors, mutant precursors, excluding those with the C98X mutation, showed stabilization of both glycosylated and nonglycosylated pre-pro-AVP (Fig. 2). Additionally, the presence of subfragments was observed.

In a similar study, Friberg et al conducted experiments in which protease and lysosomal inhibitors were applied to the COS-1 cell line after introducing mutations. The use of protease inhibitors resulted in the stabilization of proteins in both the wild-type and mutant forms. Analysis of the results revealed bands corresponding to glycosylated, nonglycosylated, and N-terminal-derived small degradation products. Their findings demonstrated that aggregate structures formed in the ER by mutant AVP precursors, expressed in fibroblasts and neuronal cells, were translocated to the cytosol through proteasomal degradation. Researchers speculated that unsecreted mutant precursors or residues could have cytotoxic effects, potentially leading to cellular damage or cell death. Interestingly, the wild-type protein also showed some degree of stabilization, indicating a decrease in protein-folding efficiency. Moreover, both mutant pre-pro-AVP and wild-type pre-pro-AVP precursors were incapable of entering the ER due of the functional activity of the signaling peptide. Consequently, they are synthesized in the cytosol and subsequently degraded by proteasomes (6).

In studies conducted in mice with errors in the ERAD mechanism, it was demonstrated that wild-type pro-AVP is a physiological substrate of ERAD, and even under normal conditions, incompletely folded small polypeptide fractions are continuously synthesized (18). However, unlike other studies that obtained similar results, our study using HA-FLAG-tagged plasmids showed that because of the capture of proteins with anti-HA antibodies, small-sized subfractions were not fully captured. Disulfide bonds, which are chemical bonds formed between 2 cysteine residues, play a crucial role in stabilizing proteins. Previous research has shown that some mutant AVP precursor proteins are degraded, whereas others accumulate and form aggregates within the ER, potentially leading to neuronal cell death. Additionally, ongoing studies have revealed that mutant AVP precursors form heterodimers with wild-type precursors, which negatively affects the transport of wild-type AVP from the ER to the Golgi apparatus. This heterodimer structure has been postulated to increase cellular cytotoxicity (5). Studies have shown that mutant precursors form a disulfide-bound homo-oligomer structure, leading to the formation of large aggregate structures with a fibrillar appearance within the ER (8). In our studies involving G45C and E108D-1 mutations, under nonreducing conditions, we obtained bands of different sizes, and two-dimensional SDS-PAGE analysis confirmed that the mutant precursors indeed formed a disulfide-bound homo-oligomer structure (Fig. 3C).

In this study, immunofluorescence and electron microscopy were performed to investigate the localization of the aggregate structures formed by mutant precursors. COS-1 cells expressing both mutant and wild-type pro-AVP were labeled with anti-AVP and anti-BAP31 antibodies (an endogenous ER membrane protein) and then visualized using various fluorescent stains. As expected, the wild-type protein, secreted from the cell, did not yield any image, and only the ER marker emitted fluorescence. However, the aggregate structures formed by mutant precursors accumulated within the ER, and the morphologies of these aggregates remained similar across different mutations. Moreover, cotransfection experiments were carried out using secretogranin II, a protein that forms granule-like structures within the cell, along with the mutant precursors. The results showed that accumulation in the ER was coexpressed with secretogranin II, and the granule structures appeared distinct from the aggregate structures (Fig. 4). A similar study showed that the expression of regulated secretory proteins, such as granins and prohormones, can lead to the formation of granule-like structures in various nonendocrine cell lines due to aggregation. In these studies using immunogold electron microscopy, the authors observed that mutant precursors developed large aggregates within the ER (7, 19).

Ito et al reported the presence of vasopressin aggregates in the ER, which is distinct from other diseases, such as Alzheimer's and Huntington's. Misfolded and unfolded proteins have been shown to cause cytotoxicity in vasopressinergic cells by trapping them in the ER. The C67X mutation, which leads to a deficiency in the C-terminal end of provasopressin, has been shown to reduce cell viability through a mechanism other than apoptosis when stably expressed in Neuro2A neuroblastoma cells (5). Similarly, immunofluorescence analyses have been conducted with heterozygous knock-in mice carrying this mutation, and these studies have indicated a gradual decrease in AVP production and an evident loss of vasopressinergic neurons (20, 21).

The results of electron microscopy analysis revealed that both wild-type and mutant precursors were labeled with a 15 nm immunogold and visualized. As expected, the mutant protein precursors accumulated within the ER lumen at different densities, leading to the disruption of the ER architecture and an expansion of the structure in the form of vesicles (Fig. 5), except for the C98X stop codon. This disruption of ER function could potentially cause ER stress, and the aggregate structures within the ER may exhibit cytotoxic properties, possibly leading to apoptosis. Similarly, in their electron microscopy studies with both COS-1 and Neuro2a cells, Birk et al demonstrated that the mutant precursors ΔE47 (A-C) and ΔG227 developed large aggregations within the ER. Furthermore, within the scope of our study, we observed that the aggregate structures formed in images taken from some cell sections were present both intracellularly and extracellularly (7).

In in vitro studies with bacteria, mutant precursors tagged with a 6-His tag were expressed, and the protein was purified. The mutant proteins were visualized using a TEM. These results demonstrate that the mutant precursors spontaneously assemble into fibrillar structures, as illustrated in Fig. 6. In our research, we previously conducted functional analysis studies on the G45C, G88V, and 207_209delGGC mutations. We used the Neuro2a cell line, transfected with wild-type and mutant constructs, to compare the levels of AVP in the supernatant. The results indicated that the levels of 207_209delG and wild-type AVP were similar, whereas the levels of G45C and G88V mutant precursors were lower. Furthermore, immunofluorescence imaging studies showed that some of the G45C and G88V mutant precursors were localized in the ER, while 207_209delGGC and wild-type precursors were distributed throughout the cytoplasm (10).

In our electron microscopy studies, we observed that the wild-type precursor was localized within the vesicles, as depicted in Fig. 5. Similarly, the 207_209delGGC mutation formed aggregate structures within the cell; interestingly, some of these aggregates were also released outside the cell, resembling the behavior of the wild-type precursor. These findings agree with the results of our detailed functional analyses.

Fost et al introduced a Glu108X (stop codon) mutation at codon 108, adding to the existing literature (22). In contrast to previous studies, we introduced 2 mutations in this research that have not been previously studied in the literature—E108D-1 (GAG->GAC) and E108D-2 (GAG->GAT) —both resulting in a single-base change. This change replaced the amino acid glutamate with aspartate in both mutations. Glutamate is known to play a crucial role in protein conformation and its tendency to form helix structures. Therefore, this change in amino acid suggests that it may lead to errors in proper folding of the protein, potentially causing misfolded proteins to lose function or form abnormal structures. Interestingly, despite being identical in amino acid change, both immunoblot and immunofluorescence studies showed that intracellular protein expression was lower for the E108D-2 mutation than E108D-1. This discrepancy in protein expression levels between the 2 mutations may be attributed to other factors or regulatory mechanisms that influence protein expression differently in each mutant. Further investigations are needed to elucidate the underlying reasons for this difference in protein expression between E108D-1 and E108D-2 mutants.

In conclusion, our study provides detailed expression analyses of various mutations associated with ADNDI, including G45C, 207_209GGCdel, G88V, C98X, C104F, E108D-1, E108D-2, and R122H. This comprehensive information significantly contributes to the existing literature. Notably, our research represents the first investigation of the E108D-1, E108D-2, and R122H mutations, adding novel insights to the field.

When reviewing the existing literature, it is evident that there are limited studies focusing on neurodegenerative diseases, including ADNDI, that are similar to our research. However, consistent results have been obtained in studies conducted by various research groups. Collectively, these findings support the idea that the accumulation of fibrillary aggregates within the ER may contribute to disease development. Furthermore, based on the evidence from our study and other related studies, it can be suggested that ADNDI belongs to a group of neurodegenerative diseases caused by disulfide-dependent homo-oligomer fibrillary amyloid aggregates.

The similarity of our findings to those of other research groups further strengthens the reliability of our conclusions. The identification of fibrillary aggregates within the ER as a potential contributor to ADNDI pathogenesis opens new avenues for understanding the underlying mechanisms and potential therapeutic targets. Our study and other similar investigations enhance the understanding of ADNDI and its position within the broader spectrum of neurodegenerative diseases, providing valuable insights for future research and potential therapeutic strategies.

## Data Availability

Some or all datasets generated during and/or analyzed during the current study are not publicly available but are available from the corresponding author on reasonable request.
